# Nonoperative management of splenic injury in closely monitored patients with reduced consciousness is safe and feasible

**DOI:** 10.1186/s13049-019-0668-5

**Published:** 2019-12-05

**Authors:** Michel Teuben, Roy Spijkerman, Taco Blokhuis, Roman Pfeifer, Henrik Teuber, Hans-Christoph Pape, Luke Leenen

**Affiliations:** 1 Department of Trauma, University Medical Centre Utrecht, Heidelberglaan 100, 3584, CX Utrecht, Suite G04.232 The Netherlands; 2 Department of Traumatology, University Hospital Zurich, Zurich, Switzerland

**Keywords:** Nonoperative management, Blunt spleen trauma, Altered mental status, GCS-score, Outcome

## Abstract

**Background:**

Treatment of blunt splenic injury has changed over the past decades. Nonoperative management (NOM) is the treatment of choice. Adequate patient selection is a prerequisite for successful NOM. Impaired mental status is considered as a relative contra indication for NOM. However, the impact of altered consciousness in well-equipped trauma institutes is unclear. We hypothesized that impaired mental status does not affect outcome in patients with splenic trauma.

**Methods:**

Our prospectively composed trauma database was used and adult patients with blunt splenic injury were included during a 14-year time period. Treatment guidelines remained unaltered over time. Patients were grouped based on the presence (Group GCS: < 14) or absence (Group GCS: 14–15) of impaired mental status. Outcome was compared.

**Results:**

A total of 161 patients were included, of whom 82 were selected for NOM. 36% of patients had a GCS-score < 14 (*N* = 20). The median GCS-score in patients with reduced consciousness was 9 (range 6–12). Groups were comparable except for significantly higher injury severity scores in the impaired mental status group (19 vs. 17, *p* = 0.007). Length of stay (28 vs. 9 days, *p* < 0.001) and ICU-stay (8 vs. 0 days, *p* = 0.005) were longer in patients with decreased GCS-scores. Failure of NOM, total splenectomy rates, complications and mortality did not differ between both study groups.

**Conclusion:**

This study shows that NOM for blunt splenic trauma is a viable treatment modality in well-equipped institutions, regardless of the patients mental status. However, the presence of neurologic impairment is associated with prolonged ICU-stay and hospitalization. We recommend, in institutions with adequate monitoring facilities, to attempt nonoperative management for blunt splenic injury, in all hemodynamically stable patients without hollow organ injuries, also in the case of reduced consciousness.

## Background

The spleen is the most frequently injured intra-abdominal organ [1, 2]. Treatment of blunt splenic injury has undergone an extensive change over the past decades [3–5]. Currently, selective nonoperative management (NOM) is the preferred treatment and is used in up to 70% of patients [6–9]. A large number of studies have validated this treatment modality with success rates exceeding 80% [8, 10, 11]. The most important prerequisite for successful NOM is adequate patient selection. Criteria for nonoperative management have expanded with increasing experience. Nowadays, the presence of hemodynamic instability is considered the only absolute contraindication for NOM [3]. However, controversy exists regarding the impact of altered mental status on outcome of NOM. Impaired mental status is a frequent problem in patients with suspected blunt splenic injury. An impairment of mental status in trauma patients is most frequently a result of shock or associated head injury. Other possible causes can be intoxication, hypoxemia, hypercarbia or a combination thereof. According to Iiterature, up to 70% of patients sustaining blunt abdominal injury sustain some degree of head injury [12, 13].

In some institutions a altered consciousness was an absolute contraindication for observational therapy in the beginning of the NOM era. lt is believed that these patients were not suitable candidates for NOM due to the inability to perform an adequate physical examination. Unreliable serial physical examination could result in missed onset of hemorrhage, and therefore delayed surgical intervention [14–16]. The last studies investigating outcome and safety of nonoperative management of blunt splenic injury in patients with altered mental status were published more than a decade ago with treatment and monitoring options having improved markedly since then [8, 17]. Furthermore, a recent literature review suggests that lower Glasgow Coma Scale (GCS)-scores are predictive for failure of NOM [18]. In our experience, improvements in diagnostics and monitoring led to improved quality and outcome of nonoperative therapy for patients with blunt abdominal injuries. Therefore, in line with current World Society of Emergency Surgery-guidelines we do not consider a GCS-score < 12 alone as a contraindication for NOM [19].

Therefore the purpose of this study was to determine whether the presence of neurological impairment affects the outcome of nonoperative management for blunt splenic injury in adult patients when monitored closely. We hypothesized that altered mental status in patients treated by nonoperative management for blunt splenic trauma is not associated with impaired outcome.

## Patients and methods

For this study our prospectively maintained trauma database was utilized. All adult patients (age > 15 years) who underwent therapy for blunt splenic injury during a 14- year period were identified. The trauma database included all patients who were admitted to our level-I-trauma centre between January 2000 and February 2014. Due to pre-hospital triage systems in our region, severely injured patients are primarily transported to our institution. In our traumaregion, stable patients without signs of craniocerebral injuries are preferably admitted to level-II-trauma centres and therefore excluded from this analysis. Patients transferred from other facilities were excluded as well.

For the purpose of this study we included all patients that were initially selected for nonoperative management. Patients who died before total diagnostic work-up was completed, as well as patients with non-survivable brain injury were excluded.

The data gathered for each patient included: demographics, mechanism of injury (MOI), Injury Severity Score (ISS), Abbreviated Injury Score of splenic injury (AIS-spleen), Glasgow coma scale, hemodynamics on admission, and outcome. Outcome analysis included mortality, failure of NOM (fNOM), complications, length of hospital stay, and intensive care unit (ICU)-days. Splenic injuries were graded according to the Abbreviated lnjury Score, which was based on computed tomography (CT) data [20].

In all hemodynamically stable patients abdominal sonography or CT scanning with intravenous contrast was performed before they were admitted to the ICU or ward.

We defined failure of NOM as the need for laparotomy in patients initially admitted to the ICU or ward for nonoperative treatment.

Nonoperative management in our institution includes initial observation on a monitored intermediate care unit or an intensive care unit, frequent examinations of vital signs and physical examinations (including abdominal and neurological examination, fluid administration and frequent hemoglobin level measurements). The aim of intravenous fluid therapy in all patients (including those with craniocerebral injury) is to maintain systolic blood pressure of at least 90 mmHg. This semi-restrictive volume policy is believed to minimize intra-abdominal hypertension and subsequent bleeding, while maintaining sufficient organ perfusion. Angio-embolization is only indicated in patients initially selected for NOM and with deteriorating hemodynamic status due to diagnosed ongoing splenic blood loss. Our protocol including restricted utilization of angio-embolization in blunt splenic trauma has previously been shown not to be associated with impaired outcome [21]. Treatment guidelines and selection criteria for non-operative management/surgical intervention/angio-embolization did not change during the study period. Decision making was performed by the attending trauma surgeon. 

Patients were divided into two groups based on presence, or absence of neurological impairment. Neurological impairment was defined as GCS-score ≤ 13. Group I therefore consisted of patients with GCS-scores 14 and 15, and group II included patients with altered mental status (GCS-score ≤ 13). GCS-score was measured for all patients upon admission. We compared morbidity and mortality between groups to determine the impact of neurological status on outcome of NOM. Complications were scored according to the Clavien-Dindo classification [22].

All procedures performed in this study are in accordance with the ethical standards of the institutional research committee and with the 1964 Helsinki declaration and its later amendments. Execution of the study was approved by the institutional review board.

All statistical analyses were performed using a commercially available statistics software package (SPSS, Version 22.0, Chicago,IL). Continuous data are expressed as median (Interquartile range) and analyzed by the Mann Whitney U Test. Categorical data were analyzed by the Fisher’s exact test. A significance Ievel of *p* < 0.05 was maintained.

## Results

During the study period, a total of 161 trauma patients with blunt splenic trauma were admitted to our level-one-trauma center. The median age of our population was 32 (22–53) years, with a male predominance of 76% (122 male vs. 39 female). The median ISS (IQR) was 25 (16–34), and 64% of patients had an GCS > 13.

**Fig. 1:**
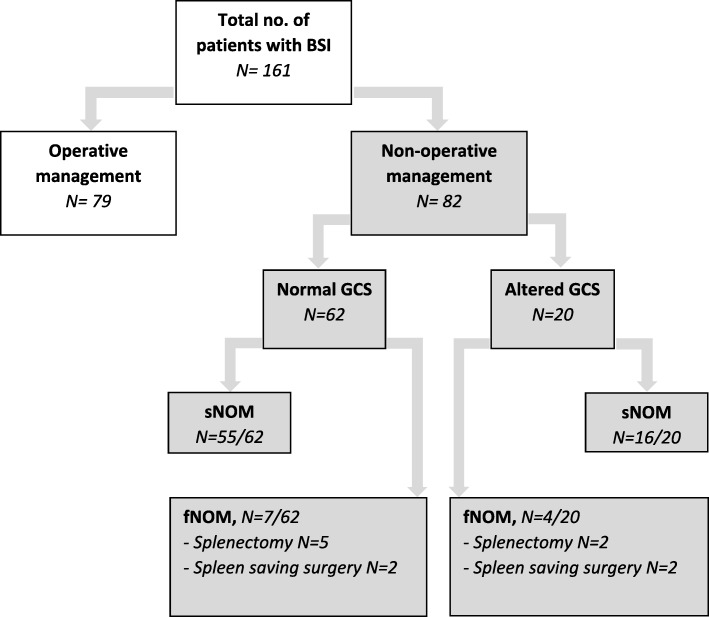
Flowchart and patient selection

As shown in Fig. 1, a total of 82 patients were treated nonoperatively for blunt splenic injury. The group consisted of 65 male and 17 female patients with a median age of 29 (range 21–51) years.

The mechanisms of injury were motor vehicle accident in 19 patients, bicycle accidents in 14 patients, motorcycle accident in 22 patients, automobile-pedestrian accident in 7 patients and other injury mechanisms in 25 patients. The median ISS (IQR) was 18 (9–27). Median systolic blood pressure on admission was 130 (range 120–140) and a median heart rate of 88 (range 76–100) beats per minute was encountered.

The Organ Injury Score of splenic injury was less than Grade IV in 66 patients, Grade IV in 11 and Grade V in 3 patients. No relevant comorbidities were found. Twenty patients had impaired mental status, of whom 2 patients were diagnosed with critical craniocerebral injuries (AIS 5) and 14 patients with AIS 3–4 craniocerebral injuries. Additionally, 4 patients had impaired GCS-scores due to the trauma severity and hypovolemic shock state. One patient had a severe head injury diagnosed as well as a positive drug test on admission. In all other patients, intoxications were ruled out.

### Impact of neurological impairment on outcome of nonoperative management

To determine the impact of neurological impairment we compared the patients with normal GCS-score and those with decreased GCS-scores. A total of 20 patients had diminished GCS. The median GCS-score (range) was 9 (6–12) and five patients had a GCS-score less than 7.

The groups were similar in baseline patient characteristics and splenic injury scores. (Table 1). As anticipated, median ISS was significantly higher in the neurologically impaired group compared with group I (29 versus 17; *p* = 0.007). Hemoglobin levels were also slightly lower in the neurologically impaired group, however statistical significance was not reached (8,4 versus 7,6; *p* = 0.105). In both groups there was a male predominance.

**Table 1:** Patient and hemodynamic characteristics in the presence or absence of neurological impairment

	Group I: Normal mental status G*CS = 14/15* (*n* = 62)	Group II: Altered mental status *GCS ≤ 13* (*n* = 20)	*P* value
Age in years	32 (23–53)	26 (20–33)	0.166
Gender (M/F)	48/14	17/3	0.545
GCS	15 (15–15)	9 (6–12)	*P* < 0.001*
AIS spleen	2 (2–3)	2 (2–3)	0.290
ISS	17 (4–24)	29 (18–34)	0.007*
Contrast blush on CT	5	2	0.700
Serum hemoglobin (mmol/l)	8.4 (7.8–8.9)	7.6 (7.1–8.4)	0.105
Systolic blood pressure (mmHg)	130 (120–145)	125 (113–140)	0.727
Pulse rate (BPM)	88 (77–100)	90 (70–103)	0.845

All data are in median (IQR):*, *p* < 0.05: Mann Whitney U test

Table 2 reviews outcome data for both neurologically impaired and non-impaired patients. Median length of stay (28 vs. 9 days; *p* < 0.001) and duration of ICU stay (8 vs. 0 days; *p* = 0.005) were significantly longer in patients with a decreased GCS-score. No differences in the failure rate of NOM and number of complications were found between the groups. In two patients, failure of NOM was prevented by angio-embolization. Failure of NOM did not occur in any patient with a contrast-blush on initial CT-scanning.

**Table 2:** Outcome of nonoperative management in the presence or absence of neurological impairment

	Group I: Normal mental status *GCS = 14/15* (n = 62)	Group II: Altered mental status *GCS ≤ 13* (n = 20)	*P*-value
Failure of NOM	7	4	0.449
*Splenectomy*	5/7	2/4	0.477
ICU-stay (days)	0 (0–2)	8 (2–25)	*p* < 0.001*
Length of hospital stay (days)	9 (7–19)	28 (13–59)	0.005*
Complications (no.)	18	12	0.085
Complication severity^a^	0 (0–2)	2 (0–2)	0.069
Mortality	0	0	*p* > 0.393

All data are in median (IQR); *,*p* < 0.05: Mann Whitney U test/Fisher Exact test. ^a^According to Clavien-Dindo-classification

Among the 20 patients with neurological impairment, NOM was successful in 16 cases (80%). In the group with regular trauma patients, NOM failed in 7 out of 62 individuals.

Failure of NOM occurred due to development of hemodynamic instability in seven patients, of whom 3 patients were neurologically impaired. Serum hemoglobin levels in these patients remained relatively unchanged over time. Nevertheless, surgical intervention was mandated in these patients due to the inadequate response to resuscitation and fluid challenge.

In three patients (including one neurologically impaired individual) a gradual progressive decrease in serum hemoglobin levels, combined with progressive sonographic intraabdominal fluid and tachycardia resulted in the need for surgical intervention. One patient with normal GCS-scores was operated on as he had gradually worsening hemodynamic parameters and deteriorating respiratory status.

Four spleen saving procedures were performed, of which two were in a patient with neurological impairment. The most frequent complications were pneumonia, extra-abdominal abscesses, and ileus. Two patients developed ileus after laparotomy. One patient with impaired mental status required re-laparotomy, in which a hemicolectomy was performed to treat a mesenterial contusion with subsequent secondary ischemia. Re-laparotomy in this patient was indicated because of persistent fever combined with an ileus. There were slightly more complications encountered in neurologically impaired patients. However, this trend did not reach statistical significance and moreover none of the complications were associated with delayed diagnosis related to impaired mental status. No differences were seen between groups, regarding the severity and impact of diagnosed complications as measured by the Clavien-Dindo-system [22]. All complications are shown in Table 3. Mortality was not seen in patients selected for nonoperative management.

**Table 3:** Complications

	Group I: Normal mental status (n = 62)	Group II: Impaired mental status (n = 20)
Pneumonia	4	6
Abscess (intra-abdominal)	2	3
Ileus	4	0
Respirarory failure	3	0
ARDS	1	2
Abscess (extra-abdominal)	0	1
Abdominal compartment syndrome	1	0
Pulmonary embolus	1	0
Sepsis	2	0
Total	18	12

## Discussion

There is clear evidence that supports non-operative management in blunt splenic injury [6, 9, 11, 15, 23]. However, controversy exists regarding the impact of altered mental status on the outcome and safety of NOM. The presence of concomitant head injury was considered a contraindication for non-operative therapy. There is concern that non-operative management in patients with altered mental status may miss early signs of intraabdominal hemorrhage that may subsequently delay surgical intervention [14–16]. We consider patients with impaired mental status as adequate candidates for non-operative management. Therefore, according to our hospital guidelines, patients with decreased GCS-scores have not been excluded from NOM during the past decade.

This study has shown that there are no differences in failure of NOM, complications due to NOM, or mortality between patients with normal mental status and those with impaired mental status. Thus, this study showed that hemodynamically stable patients with impaired mental status can safely be selected for NOM.

The findings of the current study are consistent with other series on intraabdominal solid organ injuries in which both spleen and liver injuries are analyzed [14, 24]. Archer et al. started a trend towards NOM in neurologically impaired patients in 1996. They showed, in a study with 187 observationally treated patients sustaining splenic and hepatic injury, no differences in morbidity or mortality between normal and neurologically impaired patients [24]. Furthermore, corresponding results were found by Keller et al. who studied pediatric patients with hepatic and splenic injuries [14]. These studies pooled splenic and hepatic injuries as they were based on the false assumption that splenic and hepatic injuries are comparable in treatment and clinical course. Our study exclusively valuated the impact of altered mental status on outcome of blunt splenic injury alone.

More recently, Shapiro et al. found in a study of 2327 patients sustaining kidney, liver or spleen injuries (AIS > 1), that nonoperative treatment was less likely to be initiated with worsening mental status, however, NOM in neurologically impaired patients sustaining liver, spleen or kidney injury was successful in more than 90% of cases [17].

Furthermore, the current study found similar success rates of NOM in both groups, despite higher injury severity scores in the neurologically impaired group versus the control group. This finding underlines the limited value of injury severity score calculations to determine therapy and to predict outcome of patients with blunt solid organ injuries.

Our findings are also in line with a study from Dhillon et al. on patients with both splenic and cerebral trauma selected for non-operative management. Interestingly, they found higher success rates of NOM in patients with brain injury than those patients without brain injuries. They suggested that this may be a result of management aimed to prevent secondary brain injury [25]. Those patients with concurrent brain injuries require close monitoring and therefore obtain optimal hemodynamic therapy. The injured spleen is likely to benefit from optimized hemodynamic monitoring and management as well. This effect may have contributed to the relatively good outcome in our neurologically impaired patients as well.

It has been shown that hypotension, defined as a systolic blood pressure < 90 mmHg, is associated with impaired cerebral perfusion as well as with deprived outcome in patients with traumatic brain injury [26]. During the course of the current study, our resuscitation guidelines did not change and included a semi-restrictive resuscitation protocol for all trauma cases, in which systolic blood pressure levels are maintained at a minimum of 90 mmHg. As other publications on permissive hypotension, utilize lower minimal systolic blood pressure levels (as low as 70 mmHg [27]), than we do, we decided to define our protocol as *semi*-restrictive. In our view this approach can safely be applied to all trauma patients (including those with traumatic brain injury).

The impact of hypertension on outcome is unclear und recommendations are controversial [28]. In our view, excessive use of catecholamines should be avoided in traumatic brain injury patients as this can lead to prominent variations in systolic blood pressure, which affects intracerebral capillary hydrostatic pressure and thereby may contribute to the development of cerebral edema [29–31].

Our study is limited by a relatively small sample size, although this sample size is comparable to other key publications in which data on hepatic and splenic injuries have been pooled [13]. Given the differences in therapy as well as prognosis and outcome between hepatic injuries and splenic injuries, we believe that there is a need to analyze outcome of splenic injury separately and this study is the first to do so. Furthermore, as we have used a strictly maintained prospective database, there are no missing data. Moreover, due to the detailed data provided by the database, precise information about the conditions surrounding complications, including the indications for laparotomy, were obtained. The relatively high rates of initial operative therapy and failure of NOM are most likely due to the relative trauma severity (and high trauma load) of patients admitted to our level one trauma center. This is supported by the high median ISS-score and large proportion of patients with high-grade splenic injury in the current study population.

## Conclusion

The current study shows that non-operative management for blunt splenic trauma in patients with altered mental status is a viable treatment modality in well-equipped institutions. However, the presence of neurologic impairment is associated with prolonged ICU-stay and hospitalization, likely due to the management of the neural injury and related prolonged hemodynamic monitoring itself. We therefore recommend institutions with adequate monitoring facilities, to attempt nonoperative management for splenic injury in all hemodynamically stable patients without hollow organ injuries, regardless of neurological status.

## Data Availability

The datasets analyzed during the current study are not publicly available due to privacy issues. Please contact author for data requests.

## Abbreviations

AIS: Abbreviated injury score
CT: Computed tomography
fNOM: Failure of nonoperative management
GCS: Glasgow Coma Scale
ICU: Intensive care unit
IQR: Interquartile range
ISS: Injury severity score
MOI: Mechanism of injury
NOM: Nonoperative management
